# Mesenchymal stem cell derived-exosomes: a modern approach in translational medicine

**DOI:** 10.1186/s12967-020-02622-3

**Published:** 2020-11-27

**Authors:** Sepideh Nikfarjam, Jafar Rezaie, Naime Majidi Zolbanin, Reza Jafari

**Affiliations:** 1 Department of Medical Biotechnology, Faculty of Advanced Medical Sciences, Tabriz University of Medical Sciences, Tabriz, Iran; 2 Solid Tumor Research Center, Cellular and Molecular Medicine Research Institute, Urmia University of Medical Sciences, Shafa St, Ershad Blvd, P.O. BoX: 1138, 57147 Urmia, Iran; 3 Department of Pharmacology and Toxicology, Faculty of Pharmacy, Urmia University of Medical Sciences, Urmia, Iran

**Keywords:** Mesenchymal stem cell, Exosome, Extracellular vesicle, Exosome isolation, Regenerative medicine

## Abstract

Mesenchymal stem cells (MSCs) have captured great attention in regenerative medicine for over a few decades by virtue of their differentiation capacity, potent immunomodulatory properties, and their ability to be favorably cultured and manipulated. Recent investigations implied that the pleiotropic effects of MSCs is not associated to their ability of differentiation, but rather is mediated by the secretion of soluble paracrine factors. Exosomes, nanoscale extracellular vesicles, are one of these paracrine mediators. Exosomes transfer functional cargos like miRNA and mRNA molecules, peptides, proteins, cytokines and lipids from MSCs to the recipient cells. Exosomes participate in intercellular communication events and contribute to the healing of injured or diseased tissues and organs. Studies reported that exosomes alone are responsible for the therapeutic effects of MSCs in numerous experimental models. Therefore, MSC-derived exosomes can be manipulated and applied to establish a novel cell-free therapeutic approach for treatment of a variety of diseases including heart, kidney, liver, immune and neurological diseases, and cutaneous wound healing. In comparison with their donor cells, MSC-derived exosomes offer more stable entities and diminished safety risks regarding the administration of live cells, e.g. microvasculature occlusion risk. This review discusses the exosome isolation methods invented and utilized in the clinical setting thus far and presents a summary of current information on MSC exosomes in translational medicine.

## Background

Mesenchymal stem/stromal cells (MSCs) are multipotent nonhematopoietic adult cells initially discovered by Alexander Friedenstein while studying the bone marrow. MSCs, possibly originated from the mesoderm, were reported to express CD73, CD90 and CD105 plasma membrane markers while not expressing CD14, CD34 and CD45 molecules [1, 2]. In addition to the bone marrow, MSCs can be isolated from other adult tissues including adipose tissue, amniotic fluid, dental pulp, placenta, umbilical cord blood, Wharton’s jelly and even the brain, kidney, liver, lung, spleen, pancreas and thymus [1, 3]. MSCs are known for their ability of differentiation, self-renewal and colony formation [4]. The unique capacity of MSCs to proliferate in vitro and differentiate into various cellular phenotypes represented a great opportunity for their recruitment as therapeutic agents to heal necrotic or apoptotic cells of the connective tissue. In fact, MSCs can differentiate into different lineages of mesenchymal origin including adipocytes, endothelial cells, cardiomyocytes, chondrocytes and osteoblasts as well as numerous nonmesenchymal lineages such as hepatocytes and neuron-like cells [5, 6]. The differentiation of MSCs into functional nonmesodermal cells casted doubt on the conventional paradigm that adult stem cells only differentiate from their corresponding germ layer [7]. While subsequent investigations attributed this cross-germ line differentiation to cell fusion events or methodology limitations [8, 9], the mechanism of tissue repair by MSCs particularly in nonmesodermal tissues remained to be unraveled.

It was originally assumed that upon in vivo injection, MSCs start to regenerate the damaged/diseased sites by travelling to the respective locations, engraftment, and subsequent differentiation into mature functional cells. However, this classic hypothesis was later challenged by findings from numerous animal and human studies performed during the last decades. To our surprise, it was demonstrated that MSCs neither engraft in large quantities nor for time spans long enough to explain the tissue replacement process [10]. According to a more contemporary hypothesis, MSCs employ alternate modes of tissue repair and affect their neighboring cells by inducing cell viability, proliferation and differentiation, decreasing cell apoptosis and fibrosis, stimulating extracellular matrix remodeling, and sometimes adjusting the local immune system responses to inhibit inflammation. These alternate strategies involve paracrine signaling between MSCs and the adjacent cells, which is facilitated by producing and releasing certain trophic factors, cytokines, chemokines and hormones, intercellular interactions facilitated by tunneling nanotubes, and secreting extracellular vesicles (EVs) like exosomes [3]. Exosomes derived from MSCs represent biological functions similar to these cells by contributing to tissue regeneration through enclosing and conveying active biomolecules such as peptides, proteins and RNA species to the diseased cells/tissues [11]. In this article, we overview current available exosome isolation methods intended for therapeutic application, and then summarize recent important achievements regarding the therapeutic implementation of MSC-derived exosomes in regenerative medicine in both experimental models and clinical trials.

## Exosomes

Exosomes (30–150 nm in diameter) are classified as one of the three subpopulations of EVs. The other two subpopulations include microvesicles/shedding particles and apoptotic bodies (both larger than 100 nm). Exosomes are formed by sprouting as intraluminal vesicles (ILVs) within the luminal space of late endosomes or so-called multivesicular bodies (MVBs) [12]. The ILVs are then secreted as exosomes once MVBs incorporate to the cellular membrane. Exosomes were initially detected by Rose Johnstone and colleagues in 1983 as the vesicles involved in mammalian reticulocyte differentiation and maturation [13]. Johnstone selected the term “exosome” because “the process seemed to be akin to reverse endocytosis, with internal vesicular contents released in contrast to external molecules internalized in membrane-bound structures” [14, 15]. Exosomes are constantly produced and released by numerous haematopoietic and nonhaematopoietic cell types including reticulocytes, B and T lymphocytes, platelets, mast cells, intestinal epithelial cells, dendritic cells, neoplastic cell lines, and the immune cells of the nervous system, i.e. microglia and neurons [16–18]. Accumulating knowledge has revealed that exosomes play significant role in a variety of cell-to-cell interaction pathways associated with numerous physiological and pathological functions.

According to their molecular composition and morphology, there are different populations of MVBs and thus different populations of exosomes within a cell. However, not all MVBs are destined for extracellular release. For instance, it was shown that only MVBs containing higher proportion of cholesterol could fuse with the cellular membrane of B lymphocytes and secrete exosomes [19]. More interestingly, multiple researches have shown that exosomes secreted from the apical and basolateral sides of polarized cells have different molecular compositions [20]. However, the content of exosomes partly reflects the content of their parent cells [21]. Exosomes contain a wide variety of cytoplasmic and membrane proteins including receptors, enzymes, transcription factors, extracellular matrix proteins, nucleic acids (mtDNA, ssDNA, dsDNA, mRNA and miRNA) and lipids [18]. Investigations of the exosomal protein content have revealed that some of these proteins are restricted to certain cell/tissue types, but others are common among all exosomes. While cell adhesion molecules (CAMs), integrins, tetraspanins and major histocompatibility complex (MHC) I/II proteins are common amongst all exosomes, a number of fusion and transferring proteins like Rab2, Rab7, annexins, flotillin, heat shock and cytoskeleton proteins, and MVB-generating proteins like Alix (ALG2-interacting protein X) are considered nonspecific exosomal proteins [22, 23].

Unlike proteins, exosomal lipid content is usually conserved and cell type-specific. Lipids play pivotal roles in forming and protecting exosomal structure, vesicle biogenesis and regulation of homeostasis in their target cells [24]. For instance, enhanced concentrations of lysobisphosphatidic acid in the inner phospholipid layer of MVB membrane in cooperation with Alix enable inward sprouting of MVBs and thereby exosome formation [25]. Exosomes also regulate the homeostasis of their target cells by altering their lipid composition particularly in cholesterol and sphingomyelin [25].

### Methods of exosome isolation for therapeutic application

**Fig. 1:**
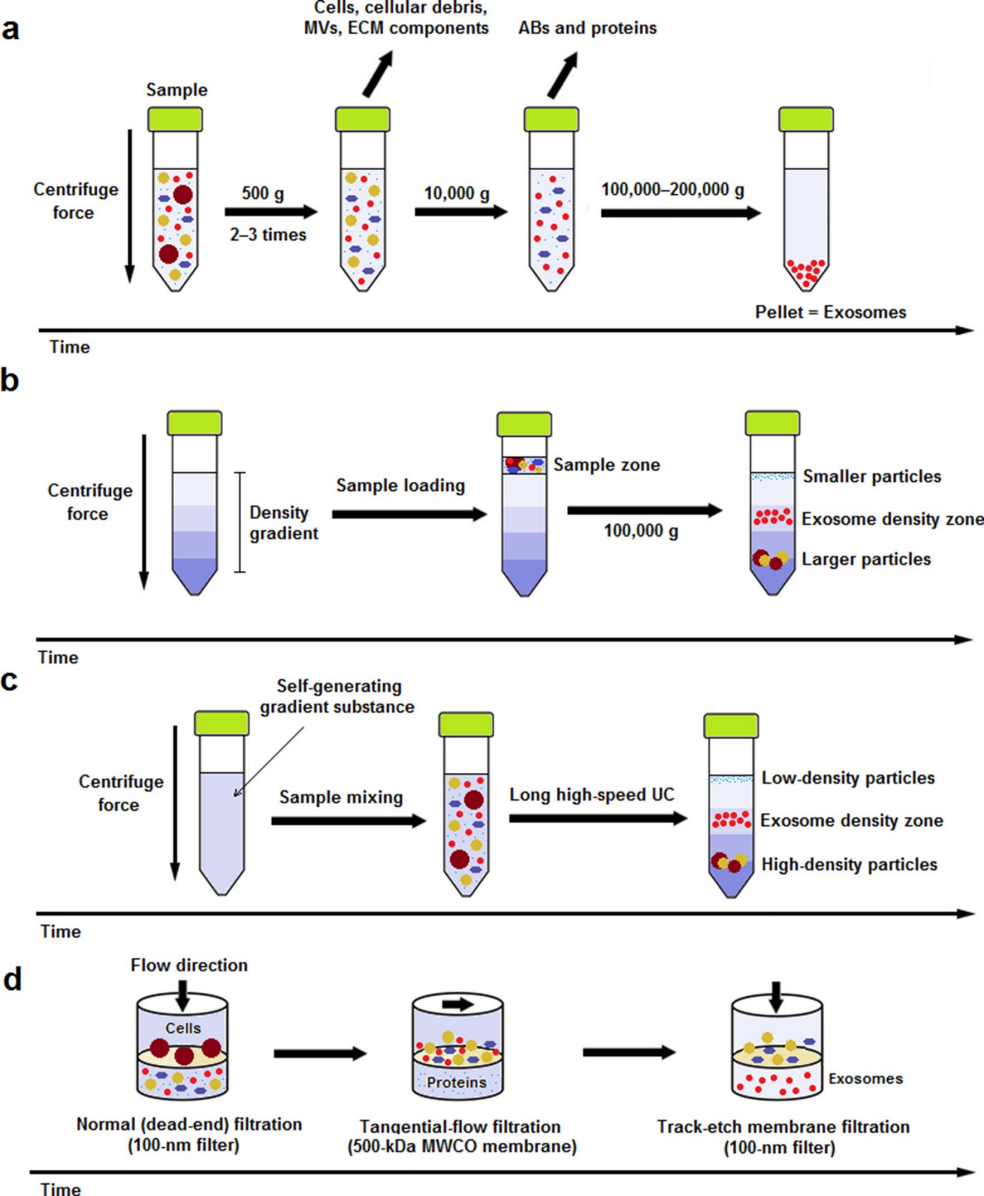
Schematic representation of most frequently utilized exosome isolation methods for therapeutic purpose. **a** Differential ultracentrifugation (DUC): Sample is subjected to 2‒3 steps of low-speed (500 *g*) centrifugation to pellet out cells, microvesicles (MVs), extracellular matrix (ECM) components, and cellular debris. The supernatant is then centrifuged at 10,000 *g* for removal of apoptotic bodies (ABs) and contaminating proteins. Finally, exosomes are retrievd by a long (60–120 min) ultracentrifugation (UC) step at 100,000–200,000 g and subsequent washing of the pellet in PBS; **b** rate-zonal ultracentrifugation (RZUC): RZUC is a type of density gradient UC (DGUC) where sample is placed at the surface of a gradient density medium such as sucrose, and following a step of UC at 100,000 g, sample components migrate through the gradient density and separate according to their size and shape; **c** isopycnic ultracentrifugation (IPUC): IPUC is another type of DGUC that separates particles based on their density. Sample is usually mixed with a self-generating gradient substance such as CsCl, and is then subjected to a long UC step. In the end, distributed components form bands, so-called the isopycnic position, where the buoyant density of the collected particles matches with the gradient density of the surrounding solution. The banded exosomes can be retrieved from the density zone between 1.10 and 1.21 g/mL by fractionation; **d** sequential filtration (SF): Sample is first subjected to a 100-nm dead-end (normal) filteration process to separate cells and larger particles. Then, contaminating proteins are excluded via tangential flow filtration using a 500-kDa MWCO membrane. Lastly, the filtrate is once more passed through a track-etch membrane filter (with pore size of 100 nm) at very low pressure in order to inhibit passing of flexible nonexosomal EVs into the filtrate while allowing for passage of exosomes

In the following section, we will discuss the two most frequently utilized methods, i.e. ultracentrifugation (UC)-based techniques and ultrafiltration (UF), for isolation of exosomes for therapeutic application. Schematic representation of the isolation methods is depicted in Fig. 1 and both methods are compared in detail in Table 1.

**Table 1:** Comparison of two most frequently utilized exosome isolation methods for clinical utility

	DUC	UF
Mechanism of exosome separation	Physical features of exosomes (size, shape and density), the exerted centrifugal force, and the viscosity of the solvent	Particle size and MWCO of the utilized filter membrane
Recovery	I	H
Purity	H	L
Specificity	I	L
Sample volume	I	H
Efficiency	I	I
Time	H	H
Cost	L	I
Complexity	I	L
Functionality of exosomes	I	I
Scalability	I	H
Advanced equipment	I	L
References	[113–117]	[118–121]

*L* low, *I* intermediate, *H* high

*Recovery*: exosomal yield; *purity*: the ability of isolating exosomes with minimum contamination; *specificity*: the ability to separate exosomes from nonexosomal content; *sample volume*: the required amount of starting material; *efficiency*: sample processing with high quality; *time*: the ability to isolate exosomes in a short amount of time; *cost*: the required amount of money; *complexity*: the need for training before use; *functionality of exosomes*: the use of isolated exosomes for downstream functional analysis without changing their efficacy; *scalability*: the ability to isolate exosomes from large sample volumes without overly increasing time, cost, or personnel required; *advanced equipment*: the need for expensive equipment and device

#### Ultracentrifugation-based techniques

When a suspension is centrifuged, its constituents will be separated on the basis of their physical features such as size, shape and density, the exerted centrifugal force, and the viscosity of the solvent. In ultracentrifugation (UC), extremely high centrifugal forces (up to 1,000,000 *g*) are applied to particulate components of a sample. UC methods are generally divided into analytical and preparative techniques. In the field of exosome isolation, preparative UC methods are considered the gold standard and account for approximately 56% of all methods employed by researchers [26]. In the following section, we will discuss two types of common preparative UC-based approaches for isolation of exosomes.

##### Differential ultracentrifugation

The successive steps of centrifugation and debris removal is referred to as differential ultracentrifugation (DUC), which is the first and still most frequent method implemented for isolation of EVs. Prior to isolation, sample is cleaned from large biocomponents and protease inhibitors are used to prevent degradation of exosomal proteins [27]. DUC consists of two to three successive low-speed (500 g) centrifugation steps to pellet out cells, microvesicles and other particles of the extracellular matrix. Further purification is then performed by 0.22 microfiltration and elimination of apoptotic bodies through centrifugation at 10,000 *g*. Finally, exosomes are retrieved by UC at approximately 100,000–120,000 *g* for 60–120 min and subsequent washing in a proper medium like phosphate buffered saline (PBS) [28]. Since the size and density of most EVs and other cellular components overlap to some extent, DUC does not yield pure exosomes, but rather results in an enrichment of exosomes. In fact, the final preparation is somewhat low in exosome recovery and often includes other particles such as serum lipoparticles [29]. If the secretory autophagy pathway is induced, lipid droplets originated from autophagosomes can also be co-isolated with exosomes [30]. The presence of large quantities of cholesteryl ester or triacylglycerol in the final preparation is defined as an index of impurity which is caused by lipoproteins or lipid droplets [31]. Therefore, it was proposed that the outcome of the 100,000 g pellet should be considered “small EVs”, not ‘exosomes’ [32]. In an attempt to increase the exosomal yield obtained by DUC, UC duration was increased to 4 h which led to serious physical damage to the exosomes, not to mention the higher contamination levels of soluble proteins [33]. DUC is laborious and time-consuming, however, it is generally applicable to large sample volumes [34], making its scalability feasible for clinical purposes [29]. Another drawback of DUC method is that its outcome is restricted by rotor capacity. Nevertheless, DUC technique requires little methodological expertise and almost no sample pretreatment [33]. Additionally, DUC is cost-effective over time and is widely utilized for isolation of exosomes in the clinical setting [35–38].

##### Density gradient ultracentrifugation

In density gradient ultracentrifugation (DGUC), a density gradient is usually constructed using iodoxinol, CsCl, or sucrose in a centrifuge tube before the separation takes place [39]. DGUC was reported to efficiently separate exosomes from soluble cellular components and protein aggregates, and resulted in the purest exosome recovery in comparison with DUC and precipitation-based techniques [40]. DGUC methods generally include rate-zonal ultracentrifugation and isopycnic ultracentrifugation. Several investigations have combined DGUC methods with DUC and reported that the purity of the separated exosomes were drastically improved. However, the gradient construction in this strategy was extremely time-consuming and further precaution was required to inhibit the gradient damage during acceleration and deceleration step [28]. DGUC usually leads to a relatively low exosomal yield and is not capable of discriminating different populations of EVs [32], which generally limits its application to large-scale exosome preparation for clinical purposes [41]. Nevertheless, several studies have successfully combined sucrose/deuterium oxide (D_2_O) DGUC with UC for isolation of exosomes for clinical use [42, 43].

*Rate-zonal ultracentrifugation*: In rate-zonal ultracentrifugation (RZUC), the sample is located in a thin zone at the surface of a shallow gradient density medium, which possesses a lower density than that of any of the sample particles [41]. Then the intended centrifugal force is exercised and the sample components start to travel through the gradient density, which gradually grows from the top to the bottom of the tube, and the particles are finally separated into various zones of the tube. Since the sample particles are denser than the gradient medium, RZUC separates components primarily based on their size and shape rather than by density [44]. The larger components and also the more spherically symmetrical particles migrate more rapidly through the gradient [44]. The duration of the centrifugation phase is of significant importance, and if not properly optimized, all particles will finally copellet at the bottom of the tube since they are all denser than the gradient [28]. To avoid exosome sedimentation, a high-density cushion is typically applied to layer the bottom of the centrifuge tube [41]. The capacity of RZUC is limited due to small loading region of the centrifuge tube which presents an obstacle for large-scale exosome preparations of clinical relevance [44].

*Isopycnic ultracentrifugation*: Isopycnic ultracentrifugation (IPUC) (also known as buoyant DUC or equilibrium DGUC) recruits the concept of buoyancy for separating particles based on their density. The sample density should be between the lowest and highest density range of the gradient [45]. In IPUC, the sample is located in a dense medium at the bottom of the gradient or uniformly mixed with a self-generating gradient substance such as CsCl [41]. Following a long high-speed centrifugation, a steep density gradient is created in the centrifuge tube [46]. As components distribute, they form bands (so-called the isopycnic position) where the buoyant density of the collected particles matches with the gradient density of the surrounding solution. The separation of exosomes into a distinct region merely depends on their density difference from all other components if a sufficient time of centrifugation is applied [41]. The banded exosomes are retrieved from the density zone between 1.10 and 1.21 g/mL by fractionation, which is performed either by removing certain amounts of fractions from the top of the tube or by draining particles with a long-needle syringe. The concentrated exosome aliquot is then subjected to a short UC at ∼100,000 g and resuspended in PBS for further analysis [41]. IPUC is a very precise technique with the ability of differentiating exosomes from other vesicles like apoptotic bodies and microvesicles as well as soluble proteins [28]. However, it is not generally applicable to clinical-scale exosome preparations [46].

#### Ultrafiltration

As is the case with any other conventional membrane filtration, ultrafiltration (UF) separates exosomes on the basis of their size and molecular weight cut-off (MWCO) of the utilized membrane filter. MWCO is an arbitrary unit representative of membrane pore size, which is utilized for characterizing UF membranes. UF membranes were initially used to purify biological fluids for retaining macromolecules particularly peptides and globular proteins. Since biological macromolecules are described by their molecular weight, the ability of UF membranes to retain these macromolecules is defined by their molecular weight. MWCO is described as the molecular weight where 90% of the macromolecular component is rejected by the membrane. Exosomes larger than pores of the membrane are held by it and smaller components are transited through the membrane. One major drawback of UF is the trapping and clogging of exosomes on membrane filter. Thus, they cannot be recovered for downstream analysis [39]. However, the isolation efficiency can be improved by starting the process with large MWCO membranes and then shifting to smaller ones [39]. UF is simpler and faster than UC, does not involve any special equipment, and can be easily scaled up and applied to the clinical field of exosomes [36, 47]. However, UF may sometimes result in exosomal damage because of the implemented shear force, which can be minimized through careful regulation of the pressure exerted on the membrane [33].

Sequential filtration (SF) is a UF technique used for isolation of exosomes by successive steps of filtration. First, the biosample is loaded on a 100-nm filter, which sieves out cells and large rigid cellular components and debris by dead-end (normal) filtration. Although their diameter is larger than 100 nm, different EV populations pass through this filter since they are flexible and soft [48]. The remaining contaminants like soluble proteins are then eliminated by tangential flow filtration using a 500-kDa MWCO membrane and the biosample is further concentrated. The filtrate is once more passed through a membrane filter, so-called track-etch membrane, with defined pore sizes (100 nm) at very low pressure in order to inhibit passing of flexible nonexosomal EVs into the filtrate while allowing for passage of exosomes. SF is one of the most efficient methods which is performed within a day. The process is automation-friendly and due to low manipulation forces, results in intact high-purity functional exosomes. Additionally, SF is capable of isolating exosomes from large sample volumes (up to 1 L) [34], which has been implemented in clinical trials [49].

### Application of mesenchymal stem cell-derived exosomes in regenerative medicine

**Fig. 2:**
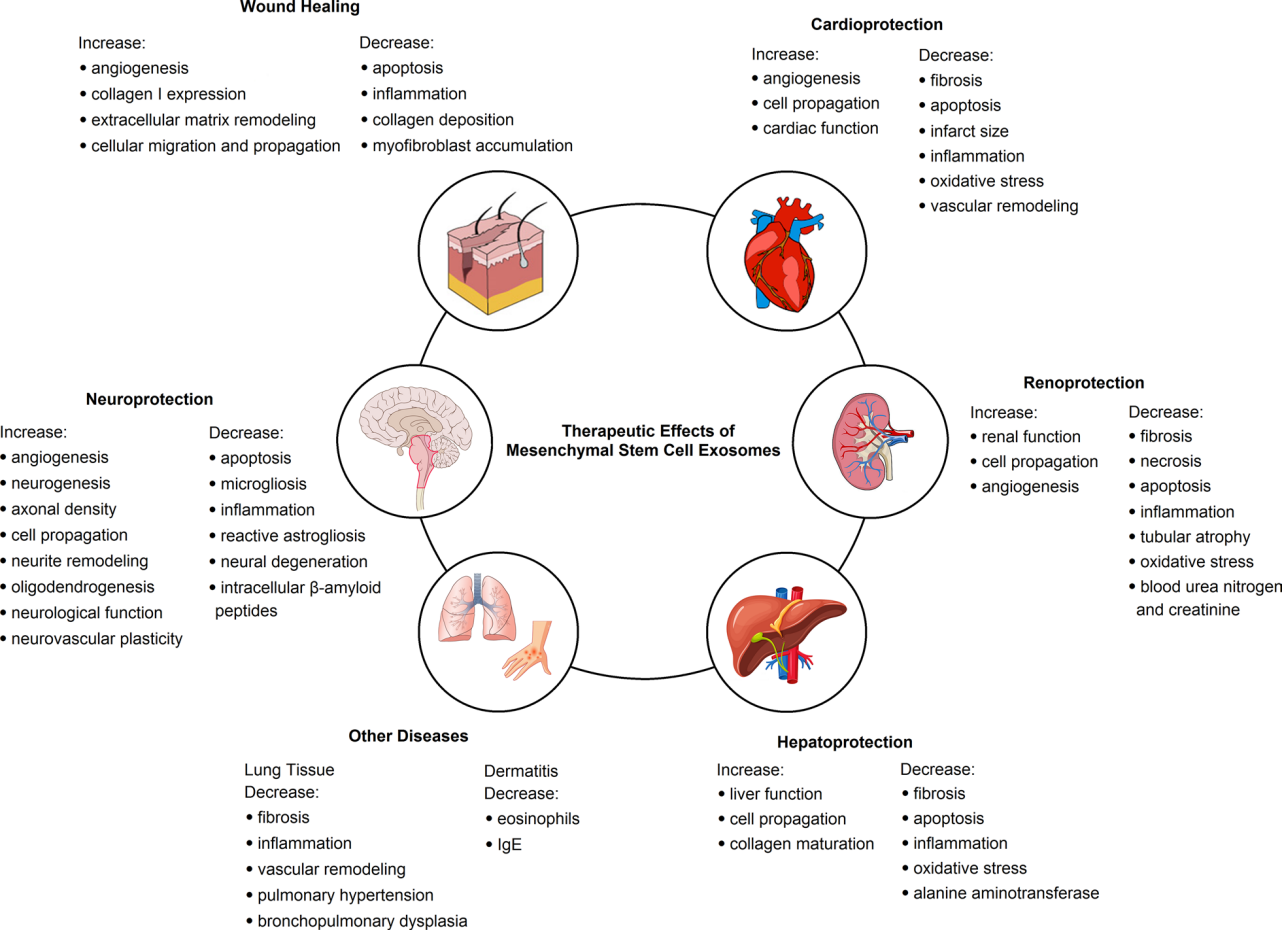
Regenerative effects of mesenchymal stem cell-derived exosomes in different diseases in preclinical experimental models

The therapeutic effects of MSC exosomes in preclinical studies are depicted in Fig. 2 and the details are summarized in Table 2.

**Table 2:** Therapeutic effects of mesenchymal stem cell-derived exosomes in different diseases in preclinical experimental models

Condition/disease	Exosome source	Experimental model	Target mechanism(s)	Therapeutic effect(s)	Ref
Cardiovascular diseases	hBM-MSC	HUVEC cell	Improved proliferation, migration, and tube formation of endothelial cells in vitro	Promoted neoangiogenesis in vitro and in vivo	[50]
		Rat MI		Improved cardiac indices, i.e. cardiac systolic/diastolic performance and blood flow	
				Reduced infarct size in vivo	
	mBM-MSC	Mouse HPH	Inactivated STAT3 pathway	Reduced vascular remodeling and HPH	[51]
			Decreased the levels of miR-17 superfamily		
			Increased miR-204 levels in lung cells		
			Repressed the hypoxic pulmonary influx of macrophages and the induction of MCP1 and HIMF		
	rBM-MSC overexpressing CXCR4	Neonatal CM	Upregulated IGF1α and pAkt levels, inhibited caspase 3, and promote VEGF expression and tubulogenesis in vitro	Increased angiogenesis	[52]
		Rat MI		Reduced infarct size	
				Improved cardiac remodeling	
	rBM-MSC	HUVEC cell	Enhanced tube formation by HUVEC cells	Decreased infarct size; preserved cardiac systolic and diastolic performance; enhanced the density of new functional capillary and blood flow recovery in vivo	[53]
		Rat MI	Compromised T cell function by impeding cell proliferation in vitro		
	mBM-MSC	Mouse MI	miR-22-enriched exosomes were secreted after MI which reduced cardiomyocyte apoptosis by direct targeting of Mecp2	Reduced infarct size and cardiac fibrosis in vivo	[55]
	rBM-MSC overexpressing GATA-4	Neonatal rat CM	miR-221-enriched exosomes reduced the expression of p53 while upregulating PUMA		[56]
			Expression of PUMA was greatly declined in CM cocultured with MSC		
	rBM-MSC overexpressing GATA-4	Neonatal rat CM	Increased CM survival, reduced CM apoptosis, and preserved mitochondrial membrane potential in vitro	Exosomal miR-19a could restore cardiac contractile function and decreased infarct size in vivo	[57]
		Rat MI	Exosomal miR-19a downregulated PTEN and triggered the Akt and ERK signaling pathways		
	mBM-MSC	HUVEC cell	Enhanced the proliferation, migration and tube formation in vitro	Promoted angiogenesis and cardiac function in vivo	[58]
		Mouse MI	The pro-angiogenic effect of exosomes is probably associated with a miR-210-Efna3 dependent mechanism		
	hEn-MSC	Neonatal CM	Overexpression and shuttling of exosomal miR-21 was attributed to suppression of PTEN, stimulation of Akt, along with Bcl-2 and VEGF upregulation	Restored cardiac function and reduced infarct size	[59]
		HUVEC cell			
		Rat MI			
	rBM-MSC	Cardiac stem cell	Triggered proliferation, migration, and angiotube formation in vitro probably mediated by a set of microRNAs	Reduced cardiac fibrosis in vivo	[60]
		Rat MI		Enhanced capillary density	
				Restored long‐term cardiac function	
Kidney diseases	hBM-MSC	mTEC	Exosomal mRNAs encoding CDC6, CDK8 and CCNB1 influenced cell cycle entry	Improved renal function and morphology	[61]
		Mouse AKI	Exosomal miRNAs regulated proliferative/anti-apoptotic pathways and growth factors (HGF and IGF1) that led to renal tubular cell proliferation		
	hAD-MSC overexpressing GDNF	HUVEC cell	Triggered migration and angiogenesis in vitro	Reduced peritubular capillary rarefaction and renal fibrosis scores in vivo	[62]
		Mouse ureteral obstruction	Conferred apoptosis resistance		
			Enhanced Sirtuin 1 signaling and p-eNOS levels		
	hBM-MSC	Rat IRI	Enhanced TEC proliferation and survival possibly via exosomal miRNA and mRNA molecules regulating renoprotective signaling routes		[63]
	Gl-MSC	Mouse IRI	Activated TEC proliferation	Ameliorated kidney function	[64]
				Reduced the ischemic damage post IRI	
	mK-MSC	HUVEC cell	Promoted cell proliferation in vitro and in vivo	Selective engraftment in ischemic tissues and significant improvement of renal function	[65]
		Mouse IRI	Improved endothelial tube formation on growth factor reduced Matrigel		
			Expressed pro-angiogenic mRNA molecules encoding bFGF, IGF1 and VEGF		
	rAD-MSC	Rat IRI	Decreased expression of TNFα, NF-κB, IL1β, MIF, PAI1, Cox2 pro-inflammatory molecules	Reduced creatinine and BUN level, and improved renal function	[66]
			Reduced the levels of NOX1, NOX2, and oxidized protein		
			Downregulated Smad3 and TGFβ fibrotic proteins		
			Enhanced Smad1/5 and BMP2 anti-apoptotic proteins		
			Upregulated CD31, vWF, and angiopoietin angiogenic biomarkers		
			Enhanced mito-Cyt C levels		
	rBM-MSC	Rat AKI	Enhanced IL10 levels	Decreased creatinine, urea, FE_Na_, necrosis, apoptosis	[67]
			Downregulated TNFα and IL6 expression		
			Increased cell proliferation		
	hWJ-MSC	HUVEC cell	Repressed NOX2 and ROS	Reduced fibrosis	[68, 69]
		NRK-52E cell	Decreased apoptosis and sNGAL levels	Improved renal function	
		Rat IRI	Enhanced cell proliferation. Upregulated Nrf2/antioxidant response element and HO1 in vitro and in vivo		
	hWJ-MSC	NRK-52E cell	Upregulated autophagy-related genes such as ATG5, -7, and LC3B in vitro and in vivo	Improved renal function in vivo	[70]
		Rat AKI	Induced mitochondrial apoptosis		
			Inhibited secretion of TNFα, IL1β, and IL6 pro-inflammatory cytokines in vitro		
	hWJ-MSC	NRK-52E cell	Reduced apoptosis and necrosis of proximal kidney tubules	Decreased BUN and creatinine levels	[71]
		Rat AKI	Decreased production of tubular protein casts through anti-oxidation and anti-apoptosis pathways in vitro and in vivo		
			Promoted cell proliferation by activating the ERK1/2 pathway		
	hBM-MSC	PTEC cell	Promoted cell proliferation by carrying IGF1 receptor mRNA, but not IGF1 mRNA		[72]
Liver diseases	hWJ-MSC	HL7702 cell	Suppressing epithelial-to-mesenchymal transition in vitro and in vivo	Reduced LF	[73]
		Mouse LF	Inactivated the TGFβ1/SMAD2 pathway		
			Alleviated hepatic inflammation and collagen deposition		
			Recovered serum AST function		
			Reduced collagen type I and III		
	hESC-MSC	TAMH, THLE-2, and HuH-7 cells	Upregulated PCNA and Cyclin D1 cell cycle proteins and anti-apoptotic Bcl-xL gene	Recovered ALI	[74]
		Mouse ALI			
	hCP-MSC	Rat LF	Exosomal miR-125b blocked Smo production and inactivated Hedgehog signaling mode	Reduced expansion of progenitors and regressed LF	[75]
	MiR‐122‐modified-hAD-MSC	Mouse LF	Exosomal miR-122 regulated the expression of IGF1R, Cyclin G1 (CCNG1) and P4HA1, which suppress HSC activation and collagen maturation	Suppressed LF development	[76]
				Reduced the serum levels of HA, P‐III‐P, ALT, AST and liver hydroxyproline content	
	mBM-MSC	Mouse ALI	Reduced pro-inflammatory cytokines and apoptosis	Decreased the serum levels of ALT and liver necrotic areas	[77]
			Upregulated anti-inflammatory cytokines		
			Triggered the number of Tregs		
	hWJ-MSC	Mouse ALI	Exosomal GPX1 cleared H_2_O_2_ and reduced apoptosis	Treated liver failure	[78]
	h/mBM-MSC	Mouse LI	Exosomal Y-RNA-1 modulated cytokine expression and reduced peripheral inflammatory responses and apoptosis	Reduced hepatic injury and increased survival	[79]
Neurological diseases	hBM-MSC	Mouse stroke	Enhanced angioneurogenesis	Recovered postischemic neurological injury	[80]
			Attenuated postischemic immunosuppression (i.e., B cell, NK cell and T cell lymphopenia) in the peripheral blood	Presented long term neuroprotection. Reduced motor coordination impairment	
	rBM-MSC	Rat stroke	Increased synaptophysin-positive regions in the ischemic boundary zone	Promoted neurovascular remodeling, axonal density and functional recovery	[81]
			Enhanced the number of newly formed doublecortin and vW		
	rBM-MSC	Rat stroke	Exosomal miR-133b decreased the expression of connective tissue growth factor and *ras* homolog gene family member A	Resulted in neurite remodeling and stroke recovery	[82]
	rBM-MSC overexpressing miR-17–92 cluster	Rat stroke	Inhibited PTEN and activated the downstream proteins, protein kinase B and glycogen synthase kinase 3β	Improved neurogenesis, neurite remodeling/neuronal dendrite plasticity and oligodendrogenesis	[83]
	hBM-MSC	Rat BI	Attenuated inflammation-induced neuronal cellular degeneration	Improved long-lasting cognitive functions	[84]
			Decreased microgliosis and prevented reactive astrogliosis		
			Restored short term myelination deficits and long term microstructural abnormalities of the white matter		
	hBM-MSC	Ewe BI	Reduced the neurological sequelae	Promoted brain function via decreasing the total number and duration of seizures	[85]
			Did not affect cerebral inflammation	Preserved baroreceptor reflex sensitivity	
	rBM-MSC	Rat TBI	Enhanced angiogenesis, the number of newborn immature and mature neurons, and decreased neuroinflammation	Improvement of spatial learning	[86]
				Recovered sensorimotor function	
	rB-MSCs	Mouse TBI	Suppressed the expression of pro-apoptotic Bcl-2-associated X protein, TNFα and IL1β	Reduced the lesion size and recovering neurobehavioral performance	[87]
			Upregulated anti-apoptotic protein B-cell lymphoma 2		
			Modulated microglia/macrophage polarization		
	rBM-MSC	Rat SCI	Regulated macrophage function by targeting M2-type macrophages in the injured sites		[88]
	rBM-MSC	Rat SCI	Reduced the proportion of A1 astrocytes via blocking the nuclear translocation of the NF-κB p65	Reduced lesion area	[89]
			Reduced the percentage of p65 positive nuclei in astrocytes and TUNEL-positive cells in the ventral horn		
			Downregulated IL1α, IL1β and TNFα		
			Increased the expression of myelin basic protein, synaptophysin and neuronal nuclei		
	hBM-MSC	Rat SCI	Showed anti-inflammatory responses in the damaged tissue and disorganization of astrocytes and microglia	Improved locomotor activity	[90]
	mBM-MSC	Mouse AD	Normoxic MSC exosomes: Decreased plaque deposition and Aβ levels	Normoxic MSC exosomes: Recovered cognition and memory impairment	[91]
			Reduced the activation of astrocytes and microglia	Preconditioned MSC exosomes: Improved learning and memory capabilities	
			Downregulated TNFα and IL1β and upregulated IL4 and IL10		
			Deactivated STAT3 and NF-κB		
			Preconditioned MSC exosomes: Reduced plaque deposition and Aβ levels		
			Upregulated growth-associated protein 43, synapsin 1, and IL10		
			Decreased the levels of glial fibrillary acidic protein, ionized calcium-binding adaptor molecule 1, TNFα, IL1β		
			Deactivated STAT3 and NF-κB		
			Enhanced miR-21 levels		
	hAD-MSC	Mouse N2a cell	Exosomes carried enzymatically active neprilysin and decreased both secreted and intracellular Aβ levels		[92]
	hDP-MSC	ReNcell VM human neural stem cell	Rescued dopaminergic neurons from apoptosis via inducing 6-hydroxy-dopamine		[93]
Wound healing	hWJ-MSC	EA.hy926 and HFL1 cells	Triggered propagation, migration, and tube formation in vitro	Improved wound healing in vivo	[94]
		Rat skin burn	Stimulated β-catenin nuclear translocation		
			Upregulated proliferating cell nuclear antigen, cyclin D3, N-cadherin, and β-catenin		
			Downregulated E-cadherin		
	hWJ-MSC	Dermal fibroblast and HEK293T cell	Exosomal miR-21, ‐23a, ‐125b, and ‐145 inhibited scar formation and myofibroblast accumulation through TGFβ2/SMAD2 pathway blockade and reduction of collagen deposition in vitro and in vivo		[95]
		Mouse skin-defect			
	hiPSC-MSC	HUVEC cell	Upregulated angiogenesis-related biomolecules	Increased microvessel density and blood perfusion	[96]
		Mouse femoral artery excision			
	hWJ-MSC	Rat skin burn	Upregulated collagen I, PCNA and CK19	Resulted in rapid in vivo re-epithelialization	[97]
			Exosomal Wnt4 contributed to β-catenin nuclear translocation and promotion of skin cell propagation and migration		
			Activated AKT pathway which reduced heat stress-induced apoptosis in vivo		
	hWJ-MSC	Rat skin burn	Decreased TNFα and IL1β levels and increased IL10 levels		[98]
			Exosomal miR-181c decreased inflammation via suppressing the TLR4 signaling route		
	hAD-MSC	HUVEC cell	Promoted angiogenesis in vitro and in vivo		[99]
		Immunodeficient mouse	Exosomal miR-125a acted as a pro-angiogenic factor by downregulating DLL4 and regulating the generation of endothelial tip cells		
	hiPSC-MSC	HUVEC cell and dermal fibroblast	Promoted collagen maturity and neoangiogenesis	Enhanced re-epithelialization	[100]
		Rat skin wound	Triggered cell proliferation and migration in vitro	Decreased scar size	
			Increased type I, III collagen and elastin mRNA expression and secretion and tube formation in vitro		
	hBM-MSC	Diabetic wound and normal fibroblasts	Promoted fibroblast propagation and migration		[101]
			Enhanced tube formation		
			Triggered Akt, ERK, and STAT3 signaling pathways		
			Upregulated HGF, IGF1, NGF and SDF1		
Other diseases	hWJ-MSC and hBM-MSC	Mouse BPD	Triggered pleiotropic effects on gene expression related with hyperoxia -induced inflammation	Relieving BPD, hyperoxia-associated inflammation, fibrosis, pulmonary hypertension and pulmonary vascular remodeling in the lung tissue	[102]
			Modulated the macrophage phenotype fulcrum, repressing the M1 state and promoting a M2-like state		
	hAD-MSC	Mouse atopic dermatitis	Decreased the levels of eosinophils, IgE, CD86^+^ and CD206^+^ cells, and infiltrated mast cells	Ameliorated atopic dermatitis in vivo	[103]
	hBM-MSC	C2C12 and HUVEC cells	Exosomal miR-494 improved angiogenesis and myogenesis in vitro and in vivo	Resulted in muscle regeneration	[104]
		Mouse muscle injury			
	hBM-MSC and hWJ-MSC	hPBMC	Enhanced the number of Tregs in vitro	Decreased educed the mean clinical score of EAE mice	[105]
		Mouse EAE			
			Decreased PBMC proliferation and levels of pro-inflammatory Th1 and Th17 cytokines inclusive of IL6, IL12p70, IL17AF, and IL22	Decreased demyelination and neuroinflammation	
			Enhanced levels of indoleamine 2,3-dioxygenase		

*Aβ* amyloid β peptide, *AD* Alzheimer’s disease, *AKI* acute kidney injury, *ALI* acute liver injury, *ALT* alanine aminotransferase, *AST* aspartate aminotransferase, *bFGF* basic fibroblast growth factor, *BPD*: bronchopulmonary dysplasia, *BMP2* bone morphogenetic protein 2, *BUN* blood urea nitrogen, *CM* cardiomyocyte, *Cox-2* cyclooxygenase 2, *DLL4* angiogenic inhibitor delta-like 4, *DP-MSC* dental pulp-derived MSC, *EAE* experimental autoimmune encephalomyelitis, *ERK* extracellular-signal-regulated kinase, *FE*_*Na*_ fractional excretion of sodium, *GDNF* glial cell line-derived neurotrophic factor, *Gl-MSC* glomeroli MSC, *GPX1* glutathione peroxidase 1, *HA* hyaluronic acid, *hCP-MSC* human chorionic plate-derived MSC, *hEn-MSC* human endometrium-derived MSC, *hESC-MSC* human emberyonic stem cell-derived MSC, *HGF* hepatocyte growth factor, *HIMF* hypoxia-inducible mitogenic factor, *hiPSC-MSC* human induced pluripotent stem cell-derived MSC, *HO1* heme oxygenase 1, *hPBMC* human peripheral blood mononuclear cell, *HPH* hypoxic pulmonary hypertension, *HSC* hepatic stellate cell, *HUVEC* human umbilical vein endothelial cell, *IGF1α* insulin-like growth factor 1α, *IL1β* interleukin 1β, *IRI* ischemia reperfusion injury, *LF* liver fibrosis, *MCP1* monocyte chemoattractant protein 1, *Mecp2* methyl CpG binding protein 2, *MI* myocardial infarction, *MIF* macrophage migration inhibitor factor, *mito-Cyt C* mitochondrial cytochrome C, *mK-MSC* mouse kidney-derived MSC, *mTEC* murine tubular epithelial cells, *NF-κB* nuclear factor κB protein, *NGF* nerve growth factor, *NOX* NADPH oxidase, *P4HA1* prolyl-4-hydroxylase α1, *PAI-1* protein expression of plasminogen activator inhibitor 1, *PCNA* proliferating cell nuclear antigen, *p-eNOS* phosphorylated endothelial nitric oxide synthase, *P‐III‐P* procollagen III‐N‐peptide, *PTEC* proximal tubular epithelial cell, *PTEN* phosphatase and tensin homolog, *PUMA* p53 upregulated modulator of apoptosis, *rAD-MSC* rat adipocyte-derived MSC, *rB-MSC* rat bone-derived MSCs, *ROS* reactive oxygen specie, *SCI* spinal cord injury, *SDF1* stromal cell-derived factor 1, *sNGAL* serum neutrophil gelatinase-associated lipocalin, *STAT3* signal transducer and activator of transcription 3, *TBI* traumatic brain injury, *TGFβ* transforming growth factor β, *TNFα* tumor necrosis factor α, *Treg* regulatory T cell, *VEGF* vascular endothelial growth factor, *vWF* von Willebrand factor

#### Cardiovascular diseases

The cardioprotective effects of exosomes secreted from MSCs was investigated in a rat myocardial infarction (MI) model using vesicles from human bone marrow-derived MSCs (hBM-MSCs). Intramyocardial injection of exosomes was reported to improve cardiac indices such as cardiac systolic and diastolic performances and blood flow [50]. MSC exosomes were also able to exert therapeutic effects by reducing vascular remodeling and hypoxic pulmonary hypertension in mice. These outcomes were mediated by inactivation of signal transducer and activator of transcription 3 (STAT3) pathway and upregulation of miR-204 in the lung cells [51]. Exosomes released from genetically modified rat BM-MSCs overexpressing CXCR4 (Exo^CR4^) were reported to enhance the levels of insulin-like growth factor 1α (IGF1α) and pAkt, inhibit caspase 3, and promote vascular endothelial growth factor (VEGF) upregulation and tubulogenesis in cultured cardiomyocytes. Moreover, when Exo^CR4^-pretreated MSC-sheet was engrafted into the damaged myocardium of a rat MI model, the infarct size remarkably decreased and angiogenesis was triggered [52]. In another study, BM-MSC exosomes promoted tube formation by endothelial cells as well as T cell inhibition, reduction of the infarct size, and recovery of cardiac systolic and diastolic performances [53, 54]. Investigation of the role of exosomal miRNA molecules demonstrated that miR-22-enriched exosomes were notably successful in decreasing the infarct size and cardiac fibrosis in a murine post-MI model via targeting MECP2 (methyl-CpG-binding protein 2) [55]. BM-MSC exosomes carrying miR-221 exhibited anti-apoptotic and cardioprotective effects by downregulating PUMA (p53 upregulated modulator of apoptosis) expression in vitro [56]. Another work performed by the same team revealed that exosomal miR-19a could reduce the infarct size and restore cardiac function through downregulating phosphatase and tensin homolog (PTEN) and triggering the Akt and ERK signaling pathways in an acute MI rat model [57]. Additionally, exosomal miR-210 were shown to promote angiogenesis and retain cardiac function both ex vivo and in vivo [58]. In an attempt to explore the cardioprotective effects of endometrium-derived MSC exosomes, exosome-mediated shuttling of miR-21 was attributed to the suppression of PTEN, stimulation of Akt, and upregulation of Bcl-2 and VEGF. As a result, cardiac function was restored and the infarct size was diminished [59]. Notable results were also found by Zhang et al. when cardiac stem cells were preconditioned with BM-MSC exosomes and administered to a rat model of MI [60]. Here, cardiac fibrosis was reduced and survival and capillary density were drastically improved.

#### Kidney diseases

In order to investigate the renoprotective effects of BM-MSC exosomes, Bruno et al. found that exosomal mRNAs encoding CDC6, CDK8 and CCNB1 along with exosomal hepatocyte growth factor (HGF) and IGF1 mediated cell cycle entry and subsequent proliferation of tubular epithelial cells while blocking apoptosis [61]. The renoprotective effects of AD-MSC exosomes overexpressing glial cell line-derived neurotrophic factor (GDNF) was investigated on renal injury using a ureteral obstruction murine model. Here, exosomes could decrease peritubular capillary rarefaction and renal fibrosis. Moreover, they stimulated angiogenesis, cell migration, sirtuin 1 signaling pathway as well as conferring apoptosis resistance [62]. In a rat model of ischemia–reperfusion injury (IRI), BM-MSC exosome administration was associated with improved tubular epithelial cell proliferation and survival [63], most probably via exosomal miRNA and mRNA molecules mediating renoprotective signaling pathways [64]. Exosomes released form kidney-derived MSCs were also recently reported to induce angiogenesis in the renal tissue by harboring pro-angiogenic mRNA molecules encoding basic fibroblast growth factor (bFGF), IGF1 and VEGF [65]. In another study on a rat model of renal IRI, adipocyte-derived MSC (AD-MSC) exosomes reduced the levels of creatinine and blood urea nitrogen (BUN) and improved renal function via downregulating pro-inflammatory cytokines and Smad3 and TGFβ fibrotic proteins as well as enhancing anti-apoptotic proteins and angiogenic biomarkers [66]. In a gentamycin-induced AKI model, administration of BM-MSC exosomes remarkably reduced inflammation by upregulating IL10 and downregulating TNFα and IL6 expression [67]. In an attempt to explore the antioxidant effects of MSC exosomes in the kidney tissue, it was revealed that exosomes derived from human Wharton’s jelly MSCs (hWJ-MSCs) could repress NADPH oxidase (NOX) and reactive oxygen species [68] while triggering Nrf2/antioxidant response element [69], which led to improved renal function and apoptosis inhibition. In a cisplatin-induced AKI model, hWJ-MSC exosomes were reported to stimulate autophagy by upregulating autophagy-related genes such as ATG5, ATG-7, and LC3B [70]. Exosomes released by hWJ-MSCs were also reported to successfully decrease BUN and creatinine levels, necrosis of proximal kidney tubules, and production of tubular protein casts through anti-oxidative and anti-apoptosis pathways [71]. Further studies have shown that when BM-MSC exosomes were co-incubated with cisplatin-injured proximal tubular epithelial cells, they were capable of promoting cell proliferation by conveying IGF1 receptor mRNA [72].

#### Liver diseases

Exosomes secreted by MSCs were also utilized in numerous studies for exploring their therapeutic effects in liver diseases. Transplantation of hWJ-MSC exosomes in a carbon tetrachloride (CCl_4_)-induced liver injury (LI) murine model was shown to limit liver fibrosis (LF) and protect hepatocytes by suppressing epithelial-to-mesenchymal transition and inactivating the transforming growth factor β1 (TGFβ1)/SMAD2 pathway [73]. Hepatoprotective effects of exosomes isolated from human embryonic stem cell-derived MSCs (hESC-MSCs) were explored in an in vitro model of acetaminophen/H_2_O_2_-induced LI and a murine model of CCl_4_-induced acute LI, and it was revealed that these exosomes contributed to tissue regeneration through upregulating the expression of PCNA and Cyclin D1 cell cycle regulators and anti-apoptotic Bcl-xL gene [74]. In a separate study using a mouse model of CCl_4_-induced LF, it was revealed that exosomes released by chorionic plate-derived MSCs harbored miR-125b that demonstrated hepatoprotective effect by blocking Smo production and thus inactivating Hedgehog signaling route [75]. Furthermore, it was revealed that AD-MSC exosomes shuttle miR-122 to hepatic stellate cells (HSCs) and regulate the expression of miR-122 target genes including IGF1R, Cyclin G1 (CCNG1) and prolyl-4-hydroxylase α1 (P4HA1), which affect cell proliferation and collagen maturation in HSCs [76]. The application of BM-MSC exosomes in a concanavalin A-induced LI (a case of immune-induced LI) could decrease the serum levels of alanine aminotransferase (ALT) and pro-inflammatory cytokines while enhancing the expression of anti-inflammatory cytokines and regulatory T cell (Treg) activity [77]. In another work, a single administration of hWJ-MSC exosomes harboring glutathione peroxidase 1 (GPX1), a vital human anti-oxidant, in a murine acute LI model could treat the disease via clearing hydrogen peroxide and relieving oxidative stress and cell death [78]. Exosomal Y-RNA-1 molecules were demonstrated to recover LI and increase survival by adjusting peripheral inflammatory responses and triggering anti-apoptosis effects in a lethal murine model of D-galactosamine/TNFα-induced liver failure [79].

#### Neurological diseases

Exosomes released from BM-MSCs were reported to exhibit therapeutic effects as they recover post-ischemic neurological injuries, enhance angioneurogenesis, and represent long-term neuroprotective functions in a murine stroke model [80]. When BM-MSC exosomes were administered intravenously to a rat stroke model, neurovascular plasticity was promoted and axonal density and synaptophysin-positive regions were improved in the ischemic margin zone of striatum and cortex [81]. Further investigations regarding BM-MSC exosomes showed that they contain miR-133b, which contributes to neurite remodeling and consequent stroke recovery upon delivery to astrocytes and neurons [82]. Additionally, it was revealed that these exosomes carry the miR-17–92 cluster, which mediates neurogenesis, neural remodeling and oligodendrogenesis in the ischemic boundary region [83]. Here, it was further demonstrated that miR-17-92 cluster-enriched exosomes have the potential of inhibiting PTEN (a confirmed target gene of miR-17-92 cluster) and consequently activating the downstream proteins, protein kinase B (mechanistic target of rapamycin) and glycogen synthase kinase 3β. In a laboratory model of inflammation-induced preterm brain injury, BM-MSC exosomes were reported to impede neural degeneration, microgliosis and inhibit reactive astrogliosis [84]. In another study, a reduction of the neurological sequelae and recovery of brain function was shown upon injection of BM-MSC exosomes [85]. While exploring the neuroprotective effects of BM-MSC exosomes in traumatic brain injury (TBI), researchers found that exosomes resulted in the promotion of angiogenesis and neuronal growth rate along with reduction of inflammation in lesion boundary zone and dentate gyrus after TBI [86]. In a separate TBI study, exosomes isolated from bone-derived MSCs (B-MSCs) exhibited neuroprotective effects by reducing the lesion size and recovering neurobehavioral performance. These outcomes were mediated by suppressing the expression of pro-apoptotic Bcl-2-associated X protein, TNFα and IL1β, upregulation of anti-apoptotic protein B-cell lymphoma 2, and modulating microglia/macrophage polarization [87]. In spinal cord injuries (SCIs), intravenously-delivered exosomes were shown to regulate macrophage functions by targeting M2-type macrophages in the injured sites [88]. Intravenous injection of BM-MSC exosomes was also reported to diminish the proportion of SCI-induced A1 astrocytes, the percentage of p65 positive nuclei in astrocytes, and the expression of IL1α, IL1β and TNFα. These mechanisms were ascribed to nuclear translocation of the NF-κB p65 [89]. Similar results were reported when systemic administration of BM-MSC exosomes showed anti-inflammatory responses in the damaged cord tissue and improved locomotor activity via disorganization of astrocytes and microglia [90]. Exosomes isolated from hypoxia-preconditioned BM-MSCs could rescue synaptic dysfunction and promote anti-inflammatory effects in an APP/PS1 murine model of Alzheimer’s disease (AD) [91]. In another study of AD, it was shown that AD-MSCs secrete exosomes containing an abundance of neprilysin, the most utilized enzyme for degradation of β-amyloid peptides in the brain tissue. The levels of secreted and intracellular β-amyloid peptides were decreased when these exosomes were transferred into neuroblastoma cells [92]. In a separate study, exosomes released from dental pulp MSCs (DP-MSCs) were reported to rescue dopaminergic neurons from apoptosis via inducing 6-hydroxy-dopamine in a 3D culture [93].

#### Wound healing

Exosomes secreted from hWJ-MSCs contribute to wound healing process via transferring Wnt4 and activating β-catenin, which leads to angiogenesis in vivo [94]. Exosomal miRNAs including miR-21, ‐23a, ‐125b, and ‐145 from hWJ-MSCs were reported to impede scar formation and myofibroblast accumulation through TGFβ2/SMAD2 pathway blockade and reduction of collagen deposition [95]. MSC exosomes were also able to trigger the expression of angiogenesis-related biomolecules and increase microvessel density and blood perfusion in the ischemic limbs of a murine model [96]. Wounds treated with hWJ-MSC exosomes demonstrated rapid in vivo re-epithelialization as well as upregulating the expression of collagen I, PCNA and CK19. Furthermore, these exosomes harbored Wnt4 that contributed to β-catenin nuclear translocation and promotion of skin cell propagation and migration [97]. MSC exosomes were also reported to ameliorate burn-induced inflammation in cutaneous wound healing. For example, hWJ-MSC exosomes exhibited anti-inflammatory effects via reducing mRNA levels of pro-inflammatory cytokines such as IL1β and TNFα while increasing IL10 levels [98]. In another study, it was shown that AD-MSC exosomes were enriched in miR-125a that acted as a pro-angiogenic factor by downregulating the angiogenic inhibitor delta-like 4 (DLL4) expression and modulating the generation of endothelial tip cells [99]. Transplantation of exosomes derived from human induced pluripotent stem cell-derived mesenchymal stem cells (hiPSC-MSCs) to the wound sites in a rat model led to rapid re-epithelialization, promoted collagen maturity, and decreased the scar size. Additionally, these vesicles triggered cell proliferation and migration and increased the secretion of type I, III collagen and elastin in a dose-dependent manner in vitro [100]. Exosomal miR-181c contributed to the suppression of TLR4 signaling route. Here, exosomes derived from BM-MSCs could dose-dependently promote fibroblast propagation and migration, tube formation, trigger Akt, ERK, and STAT3 signaling pathways, and upregulate HGF, IGF1, nerve growth factor (NGF) and stromal cell-derived factor 1 (SDF1) expression [101].

#### Other diseases

In the lung, exosomes isolated from WJ-MSCs demonstrated remarkable therapeutic effects by relieving bronchopulmonary dysplasia, hyperoxia-associated inflammation, fibrosis, pulmonary hypertension and pulmonary vascular remodeling through adjusting the phenotype of lung macrophages [102]. In a murine atopic dermatitis model, AD-MSC exosomes could decrease the levels of eosinophils, IgE, CD86^+^ and CD206^+^ cells, and the infiltrated mast cells [103]. Exosomal miR-494 contributed to muscle regeneration via improving angiogenesis and myogenesis [104]. Study of BM-MSC exosomes in an experimental autoimmune encephalomyelitis model of multiple sclerosis revealed that they were capable of downregulating pro-inflammatory cytokines and inducing Tregs [105].

### Mesenchymal stem cell-derived exosomes in clinical trials

Preclinical data have proven the safety of exosome therapy and scalability of their isolation methods from MSCs for clinical application. However, the use of MSC exosomes in clinical setting is limited due to the lack of established cell culture conditions and optimal protocols for production, isolation and storage of exosomes, optimal therapeutic dose and administration schedule, and reliable potency assays to evaluate the efficacy of exosome therapy [106–108]. There are numerous studies investigating the efficiency of MSC exosomes in the clinical settings. Although most of the clinical trials are in the recruitment and active phases, some of them have completed without publishing their results. Kordelas et al. tested the therapeutic effects of BM-MSCs in patients with steroid refractory graft-versus-host disease and found that the secretion of IL1β, TNFα, and IFNγ by PBMCs were remarkably reduced following the third exosome application [109]. In line with the ameliorated pro-inflammatory response of the PBMCs, the disease symptoms improved significantly shortly after the MSC exosome therapy started. In another study, Nassar et al. showed that the application of UC-MSC exosomes led to overall improvement in renal function in patients suffering from grade III-IV chronic kidney disease [110]. Here, exosome therapy resulted in remarkable improvement of plasma creatinine level, estimated glomerular filtration rate, blood urea and urinary albumin creatinine ratio. Furthermore, serum levels of IL10 and TGFβ1 were increased while serum levels of TNFα were decreased. There are also other ongoing trials performed to determine the safety and effectiveness of human MSC exosomes in treatment of tissue injuries which are summarized in Table 3.

**Table 3:** Mesenchymal stem cell-derived exosomes in clinical trials (https://www.clinicaltrials.gov/)

Organ	Condition/disease	Trial ID/Ref	Phase	Status	Source of exosomes	Dose/frequency/route	Location
Lung	Healthy	NCT04313647	I	Recruiting	AD-MSC	1× level: 2.0 × 10^8^/3 ml	China
						2× level: 4.0 × 10^8^/3 ml	
						4× level: 8.0 × 10^8^/3 ml	
						6× level: 12.0 × 10^8^/3 ml	
						8× level: 16.0 × 10^8^/3 ml	
						10× level: 20.0 × 10^8^/3 ml	
						All experiments: once; aerosol inhalation	
	SARS-CoV-2 pneumonia	NCT04276987	I	Completed	AD-MSC	2.0 × 10^8^/3 ml	China
						Once a day during 5 days	
						Aerosol inhalation	
		NCT04491240	I, II	Enrolling by invitation	MSC	Procedure 1: 0.5–2 × 10^10^/3 ml	Russia
						Procedure 2: 0.5–2 × 10^10^/3 ml	
						All experiments: twice a day during 10 days; inhalation	
	Bronchopulmonary dysplasia	NCT03857841	I	Recruiting	BM-MSC	20 pmol phospholid/kg	
						60 pmol phospholid/kg	
						200 pmol phospholid/kg	
						All experiments: intravenous injection	
Skin	Dystrophic epidermolysis bullosa	NCT04173650	I, II	Not yet recruiting	BM-MSC	AGLE-102 exosomes	USA
						Once a day during 60 days	
						Applied topically to the entire body	
	Chronic ulcer	NCT04134676	I	Completed	WJ-MSC	Conditioned medium gel	Indonesia
						Every week for 2 weeks	
						Applied topically to the wound	
Brain	Acute ischemic stroke	NCT03384433	I, II	Completed	BM-MSC	200 µg total protein of miR-124-loaded exosomes	Iran
						One month after attack	
						Stereotactic guidance	
	Alzheimer’s disease	NCT04388982	I, II	Not yet recruiting	AD-MSC	Low dosage group: 5 μg exosome/1 ml	China
						Mild dosage group: 10 μg exosome/1 ml	
						High dosage group: 20 μg exosome/1 ml	
						All experiments: twice a week during 12 weeks; nasal drip	
Eye	Macular holes	NCT03437759	Early phase I	Recruiting	UC-MSC	20–50 µg exosome/10 μl	China
						Single dose	
						Directly injected around macular hole area	
	Dry eye	NCT04213248	I, II	Recruiting	UC-MSC	10 µg exosome/drop	China
						4 times a day during 14 days	
						Eye drops	
Other organs/tissues	Multiple organ failure	NCT04356300	Not applicable	Not yet recruiting	UC-MSC	150 mg exosome	China
						Once a day during 14 days	
						Intravenous injection	
	Diabetes mellitus type 1	NCT02138331	II, III	Unknown	UC-MSC	First dose: Intravenous injection of exosomes isolated from the supernatant produced from 1.22–1.51 × 10^6^ MSCs/kg	Egypt
						Second dose: 7 days after the first dose; intravenous injection of MVs isolated from the supernatant produced from the same dose of MSCs utilized in the first injection	
	Osteoarthritis	NCT04223622	I	Not yet recruiting	AD-MSC	Osteochondral explants from arthroplasty patients treated with AD-MSC secretome (either complete conditioned medium or EVs)	Italy
	Graft-versus-host disease	[109]	–	Concluded	BM-MSC	1.3–3.5 × 10^10^ exosome/unit; 0.5–1.6 mg/unit (The yield of an EV fraction isolated from supernatants of 4 × 10^7^ MSCs was defined as one unit.)	Germany
						First dose: a tenth of a unit	
						Second dose: 2 days after the first dose, unit amounts were progressively enhanced and administered every 2–3 days until 4 doses	
	Chronic kidney disease	[110]	II, III	Concluded	UC-MSC	100 μg of total EV protein/kg	Egypt
						2 doses (1 week apart)	
						First dose: intravenous injection	
						Second dose: infused into the renal artery	

## Concluding remarks

Exosomes secreted by MSCs are now being extensively exploited to develop novel regenerative strategies for numerous diseases since they convey most of the therapeutic properties of MSCs. Exosomes offer a possibility of cell-free therapy, which minimizes safety concerns regarding the administration of viable cells. In many cases, the regenerative effect of MSC exosomes has been ascribed to their anti-inflammatory function in the recipient cells. Exploiting these immunomodulatory effects allows for the use of MSC-derived exosomes to treat different inflammatory and autoimmune diseases. The function of exosomes can be readily adjusted via preconditioning of MSC culture, for instance by addition of chemical factors or cytokines, exerting hypoxic conditions, and introducing gene modifications such as the CRISPR/Cas9 technology [111]. However, details about the functional mechanisms of exosomes in MSCs and their target cells continue to be elucidated. Moreover, there are still a few unresolved concerns before bringing MSC-derived exosomes into the clinical setting. Standards and guidelines should be established for vesicle size, purity, expression of certain surface biomarkers (e.g. CD9, CD63, CD81), and acceptable contamination levels for identification and quality control of the isolated exosomes. It was confirmed that the physiological state of MSCs influence the therapeutic efficiency of isolated exosomes [108]. This issue can be partly resolved by MSC preconditioning or extracting exosomes from induced pluripotent stem cells or embryonic stem cells [112] in order to diminish the lot-to-lot variation regarding primary naive MSCs. In summary, findings from various research works imply that MSC-derived exosomes possess promising therapeutic capacity for treatment of a variety of diseases. Efforts directed toward determining standards on the therapy efficacy and safety issues will speed up clinical implementation of MSC-derived exosomes as regenerative agents.

## Data Availability

Not applicable.

## Abbreviations

AD: Alzheimer’s disease
AD-MSC: Adipocyte-derived mesenchymal stem cell
AKI: Acute kidney injury
Alix: ALG2-interacting protein X
bFGF: Basic fibroblast growth factor
B-MSC: Bone-derived mesenchymal stem cell
CAM: Cell adhesion molecule
CCl_4_: Carbon tetrachloride
CRISPR: Clustered regularly interspaced short palindromic repeats
DLL4: Delta-like 4 protein
DGUC: Density gradient ultracentrifugation
DP-MSC: Dental pulp-derived mesenchymal stem cell
DUC: Differential ultracentrifugation
EV: Extracellular vesicle
GPX1: Glutathione peroxidase 1
hBM-MSC: Human bone marrow-derived mesenchymal stem cell
hESC-MSC: Human embryonic stem cell-derived mesenchymal stem cell
HGF: Hepatocyte growth factor
HSC: Hepatic stellate cell
hUC-MSC: Human umbilical cord blood-derived mesenchymal stem cell
hWJ-MSC: Human Wharton’s jelly (umbilical cord matrix)-derived mesenchymal stem cell
IgE: Immunoglobulin E
IGF1α: Insulin-like growth factor 1α
IL1β: Interleukin 1β
ILV: Intraluminal vesicle
IPUC: Isopycnic ultracentrifugation
IRI: Ischemia–reperfusion injury
K-MSC: Kidney-derived mesenchymal stem cell
LF: Liver fibrosis
LI: Liver injury
MECP2: Methyl-CpG-binding protein 2
MHC: Major histocompatibility complex
MI: Myocardial infarction
MSC: Mesenchymal stem/stromal cell
MVB: Multivesicular body
MWCO: Molecular weight cut-off
NADPH: Nicotinamide adenine dinucleotide phosphate
NGF: Nerve growth factor
P4HA1: Prolyl-4-hydroxylase α1
PBS: Phosphate buffered saline
PTEN: Phosphatase and tensin homolog
PUMA: P53 upregulated modulator of apoptosis
RBC: Red blood cell
RZUC: Rate-zonal ultracentrifugation
SCI: Spinal cord injury
SDF1: Stromal cell-derived factor 1
SF: Sequential filtration
STAT3: Signal transducer and activator of transcription 3
TBI: Traumatic brain injury
TGFβ1: Transforming growth factor β1
TNFα: Tumor necrosis factor α
UF: Ultrafiltration
UC: Ultracentrifugation
VEGF: Vascular endothelial growth factor
